# Role of Mitochondrial Iron Uptake in Acetaminophen Hepatotoxicity ^†^

**DOI:** 10.3390/livers4030024

**Published:** 2024-07-30

**Authors:** Jiangting Hu, Anna-Liisa Nieminen, Zhi Zhong, John J. Lemasters

**Affiliations:** 1 Center for Cell Death, Injury & Regeneration, Medical University of South Carolina, Charleston, SC 29425, USA; 2 Department of Drug Discovery & Biomedical Sciences, Medical University of South Carolina, Charleston, SC 29425, USA; 3 Hollings Cancer Center, Medical University of South Carolina, Charleston, SC 29425, USA; 4 Department of Biochemistry & Molecular Biology, Medical University of South Carolina, Charleston, SC 29425, USA

**Keywords:** acetaminophen, iron, mitochondria, NAPQI, •OH, oxidative stress

## Abstract

Overdose of acetaminophen (APAP) produces fulminant hepatic necrosis. The underlying mechanism of APAP hepatotoxicity involves mitochondrial dysfunction, including mitochondrial oxidant stress and the onset of mitochondrial permeability transition (MPT). Reactive oxygen species (ROS) play an important role in APAP-induced hepatotoxicity, and iron is a critical catalyst for ROS formation. This review summarizes the role of mitochondrial ROS formation in APAP hepatotoxicity and further focuses on the role of iron. Normally, hepatocytes take up Fe^3+^-transferrin bound to transferrin receptors via endocytosis. Concentrated into lysosomes, the controlled release of iron is required for the mitochondrial biosynthesis of heme and non-heme iron-sulfur clusters. After APAP overdose, the toxic metabolite, NAPQI, damages lysosomes, causing excess iron release and the mitochondrial uptake of Fe^2+^ by the mitochondrial calcium uniporter (MCU). NAPQI also inhibits mitochondrial respiration to promote ROS formation, including H_2_O_2_, with which Fe^2+^ reacts to form highly reactive •OH through the Fenton reaction. •OH, in turn, causes lipid peroxidation, the formation of toxic aldehydes, induction of the MPT, and ultimately, cell death. Fe^2+^ also facilitates protein nitration. Targeting pathways of mitochondrial iron movement and consequent iron-dependent mitochondrial ROS formation is a promising strategy to intervene against APAP hepatotoxicity in a clinical setting.

## 1. Introduction

### 1.1. Epidemiology of Acetaminophen Hepatotoxicity

Acetaminophen (also known as Tylenol^®^, paracetamol, and N-acetyl-para-aminophenol and commonly abbreviated for the latter as APAP) is one of the most used antipyretic and analgesic medications and is often combined with cough-and-cold remedies and narcotic pain relievers. APAP is generally very safe in therapeutic doses. However, an overdose of APAP causes severe liver injury, leading to elevations of serum transaminases (ALT and AST), hepatic necrosis, and even acute liver failure requiring liver transplantation [1]. APAP hepatotoxicity is the leading cause of acute liver failure in the United States, and up to 50% of cases are unintentional [2]. The currently recommended maximal therapeutic dose is 4 g/day. However, it is estimated that 6% of adults in the USA are taking over 4 g/day due to APAP combination medications [3].

### 1.2. Metabolism of APAP

At therapeutic doses in humans, 85–90% of APAP becomes conjugated with sulfate and glucuronide and is excreted in urine. Only a small portion of APAP is metabolically activated by cytochrome P450 enzymes (mainly CYP2E1) to the toxic and reactive metabolite, *N*-acetyl-*p*-benzoquinoneimine (NAPQI). Under normal conditions, NAPQI is efficiently detoxified by conjugation with glutathione (GSH) [4]. After an overdose of APAP, the sulfate and glucuronide pathways become saturated, and CYP450 produces relatively more NAPQI. Subsequently, GSH becomes depleted by conjugation with NAPQI, and additional NAPQI can no longer be detoxified, which then leads to liver damage [5,6].

### 1.3. Risk Factors of APAP Hepatotoxicity

APAP toxicity shows a threshold dose dependence such that therapeutic doses are completely non-toxic, but the threshold dose causing liver damage varies between individuals. Not all individuals with APAP overdose progress to acute liver failure. Moreover, even at a therapeutic dose, APAP hepatotoxicity can occur under certain conditions. Accordingly, the safe upper limit of APAP for therapeutic indications remains controversial [7–9]. Genetic variation within the CYP450 system can cause differing sensitivity to APAP hepatotoxicity, as well as to other risk factors [10,11].

Malnutrition, fasting, and chronic liver disease may increase the risk of APAP hepatotoxicity by decreasing hepatic levels of GSH. A 6 h fast depletes hepatic GSH levels in mice by 44% [12]. Patients with already low GSH stores as a result of fasting or malnutrition can develop severe hepatotoxicity at recommended doses of APAP [13]. Infants and adults who are alcoholic or who take certain CYP450-inducing drugs may also be more prone to liver injury from APAP [14–16]. Commonly used upregulating CYP450 drugs include rifampin, isoniazid, and phenobarbital. Chronic alcohol use also causes CYP450 enzyme induction with the increased toxic metabolism of APAP to NAPQI and enhanced hepatotoxicity, even at therapeutic doses. Fibrates, nonsteroidal anti-inflammatory drugs (NSAIDs), and alcohol are associated with a higher incidence of death in patients with APAP-associated liver injury [17]. Nonalcoholic fatty liver disease (NAFLD), recently renamed metabolic dysfunction-associated steatotic liver disease (MASLD) [18], is also associated with increased CYP2E1 activity and is accompanied by an increased risk of APAP-induced hepatotoxicity [19].

### 1.4. Treatment for APAP Hepatotoxicity

Early diagnosis means early intervention, which is crucial to prevent APAP-induced acute liver failure (ALF). *N*-acetylcysteine (NAC) is the preferred antidote for APAP toxicity. NAC prevents hepatotoxicity by replenishing GSH stores, binding with NAPQI, and enhancing sulfate conjugation [20]. NAC may further limit APAP toxicity through antioxidant and anti-inflammatory effects. For maximal protection against liver injury, NAC should be given within 8 h after an APAP overdose in patients whose plasma APAP levels are above the “possible hepatic toxicity” line of the Rumack–Matthew nomogram [21,22]. NAC can be given intravenously or by mouth with similar efficacy for improving outcomes in APAP overdoses [23]. However, the indications and dosage for NAC are debated. Other treatments include activated charcoal and liver transplantation. Activated charcoal can be used within 4 h after taking APAP to limit the gastrointestinal absorption of APAP [24]. However, this treatment is ineffective in most cases because of the rapid absorption of APAP. Liver transplantation is the ultimate treatment for patients with ALF [25].

## 2. Role of Mitochondria in Pathogenesis of APAP Hepatotoxicity

The toxic metabolite NAPQI, rather than APAP itself, causes hepatotoxicity [26]. The main mechanism causing liver injury is thought to be covalent NAPQI protein adduct formation, which leads to mitochondrial dysfunction, oxidative stress due to GSH depletion by conjugation with NAPQI, and cell death [27].

### 2.1. Mitochondrial Permeability Transition in APAP Hepatotoxicity

Mitochondria are a primary target of NAPQI. The expression of some CYP2E1 in the mitochondrial inner membrane rather than the endoplasmic reticulum may account, at least in part, for mitochondrial NAPQI protein adduct formation [28–30]. Mitochondrial protein adduct formation with NAPQI causes oxidative stress, which leads to various mitochondrial dysfunctions, including respiratory inhibition, decreased hepatic ATP, decreased mitochondrial membrane potential (ΔΨ), and the onset of the mitochondrial permeability transition (MPT) [31,32]. Interestingly, low-dose APAP, which does not cause necrosis in vivo, can still produce MPT-dependent mitochondrial depolarization, which is reversible [33,34].

The MPT is an abrupt increase in the permeability of the mitochondrial inner membrane to molecules of less than about 1500 Daltons in molecular weight [35,36]. Ca^2+^ activates MPT onset, whereas cyclosporin A (CsA) and non-immunosuppressive analogs like NIM811 inhibit permeability transition (PT) pore opening [37,38]. In one model, PT pores are formed by the voltage-dependent anion channel (VDAC) in the outer membrane, the adenine nucleotide translocator (ANT) in the inner membrane, and cyclophilin D (CypD) in the matrix. However, the genetic deletion of ANT1/ANT2 and VDAC does not prevent the onset of the MPT [39–41], although more recent studies in triple ANT1, 2, and 4 and CypD-deficient mice and cell lines indicate that the MPT requires ANT and CsA-binding CypD [42,43]. Other studies suggest that dimers or oligomers of the mitochondrial F_1_Fo-ATP synthase or the c-rings of the F_O_ subunit of the synthase form PT pores [44–46], but other studies show that Ca^2+^-induced PT pore-opening persists after genetic interventions that prevent assembly ATP synthase monomers, dimers, or oligomers [47–49]. Another recent study concludes that the ATP synthase is a negative rather than a positive regulator of PT pores [50]. In addition, regulated and unregulated conductance modes for PT pores have been described: one activated by Ca^2+^ and inhibited by CsA and the other not requiring Ca^2+^ for activation and not inhibited by CsA [51]. Consistent with regulated and unregulated pore opening, a different model of pore formation and gating proposes that PT pores are created by misfolded integral membrane proteins damaged by oxidants and other stresses. These misfolded proteins aggregate at exposed hydrophilic surfaces within the membrane bilayer to form aqueous channels. Chaperone-like proteins, including CypD, a peptidyl-prolyl cis-trans isomerase or foldase, initially block conductance through these misfolded protein clusters. However, increased Ca^2+^ acting on CypD opens these regulated PT pores, which is an effect blocked by CsA. When protein clusters exceed chaperones available to block conductance, unregulated pore opening occurs [51,52]. Thus, in this proposal, PT pores comprise multiple different molecular species, which is a conclusion increasingly made in experimental studies [42,53–55]. Nonetheless, the precise molecular composition of the PT pore or pores remains controversial.

CsA specifically blocks the MPT by binding to CypD [56]. NIM811 (N-methyl-4-isoleucine cyclosporin) is a non-immunosuppressive derivative of CsA that inhibits the MPT equivalently to CsA in isolated mitochondria [38,57]. NIM811 is protective to cultured hepatocytes and livers in vivo after a variety of injurious stresses, including ischemia/reperfusion injury, transplantation, massive hepatectomy, and cholestatic injury [58–61]. CsA and NIM811 also inhibit the MPT and attenuate APAP hepatotoxicity both in vivo and in vitro [31,33,62,63]. As discussed above, PT pores have two open conductance modes—a Ca^2+^-activated and CsA-sensitive regulated mode associated with early PT pore opening and an unregulated mode occurring later, which does not require Ca^2+^ and is not inhibited by CsA [51]. In cultured mouse hepatocytes, CsA and NIM811 delay but do not prevent APAP-induced mitochondrial depolarization, indicating that APAP initially induces a regulated MPT that is later superseded by an unregulated MPT [31]. Ultimately, the release of proapoptotic mitochondrial proteins, together with the cessation of ATP production, leads to cell death [31,64,65].

### 2.2. Apoptosis and Necrosis in APAP Hepatotoxicity

Whether apoptosis or necrosis is the major mode of cell death in APAP hepatotoxicity has been a controversial topic. The MPT plays an important role in the development of both necrotic and apoptotic cell death [66]. Specifically, the uncoupling of oxidative phosphorylation after the MPT causes ATP depletion, which leads to necrotic cell killing, whereas the mitochondrial outer membrane rupture after MPT-induced mitochondrial swelling causes cytochrome *c* release and apoptosis. In vitro, APAP mainly induces necrosis in cultured mouse hepatocytes. However, apoptosis increases when necrotic cell death is blocked [67]. Animal studies suggest that APAP-induced hepatic damage is predominantly oncotic necrosis rather than apoptosis [68]. Although modest caspase activation resulting from the release of mitochondrial proteins may occur after APAP, it is insufficient to actually cause significant apoptotic cell death [69]. Nonetheless, a human study reported increased serum apoptotic markers in patients with APAP-induced acute liver failure and suggested the predictive role of apoptotic markers in the progression of acute liver failure after APAP overdose [70].

### 2.3. c-Jun N-Terminal Protein Kinase Activation in APAP Hepatotoxicity

In mice and cultured mouse hepatocytes after APAP exposure, c-Jun N-terminal protein kinase (JNK), a mitogen-activated protein kinase (MAPK), becomes phosphorylated, signifying activation [71]. Phospho-JNK (p-JNK) then translocates to mitochondria by binding and phosphorylating the outer membrane protein SAB, an abbreviation for the SH3 domain-binding protein that preferentially associates with Bruton’s tyrosine kinase [72,73]. The subsequent release of protein tyrosine phosphatase nonreceptor type 6 (PTPN6) from SAB in the intermembrane space leads to the dephosphorylation of mitochondrial tyrosine-protein kinase c-SRC [74]. Decreased phospho-c-SRC leads to the inhibition of the respiratory chain, which enhances the generation of reactive oxygen species (ROS) [73,75]. The amplified oxidant stress then causes sustained JNK activation and promotes an APAP-induced MPT [32,76]. Platanosides, a botanical drug combination, decrease liver injury from APAP overdose in mice, possibly by preventing sustained JNK activation [77]. After low-dose APAP is given to mice, reversible hepatic mitochondrial dysfunction occurs associated with transient JNK activation [33].

## 3. Role of Oxidative Stress in APAP Hepatotoxicity

Oxidative stress is a principal mediator of toxicity and has been suggested as an important mechanism in APAP-induced hepatotoxicity. ROS formation increases after APAP exposure and agents that augment antioxidant defenses and scavenge ROS protect against APAP toxicity in vitro and in vivo [78]. The formation of ROS like O_2_•^−^ occurs selectively in mitochondria after the initial metabolism of APAP and originates at least in part from Complex III of the respiratory chain [79–82].

The Fenton or iron-catalyzed Haber–Weiss reaction is critical following oxidative stress during APAP toxicity [83]. Initially, superoxide (O_2_•^−^) may be formed by activated NADPH oxidase, loosely coupled CYP2E1, and the NAPQI-dependent disruption of the mitochondrial respiratory chain. Dismutation catalyzed by superoxide dismutase (SOD) converts O_2_•^−^ to H_2_O_2_. After an APAP overdose, H_2_O_2_ cannot be completely detoxified by glutathione peroxidase since its cofactor, GSH, becomes depleted by NAPQI. O_2_•^−^ also reduces ferric iron (Fe^3+^) to ferrous iron (Fe^2+^). Fe^2+^, thus, formed reacts rapidly with H_2_O_2_ to form the highly reactive hydroxyl radical (•OH) [27,81,83]. •OH, in turn, damages protein and DNA, as well as causing lipid peroxidation and the breakdown of membranes. However, the most critical effect of this oxidative stress is the induction of the MPT, which produces bioenergetic failure and, ultimately, cell death [31,63].

## 4. Iron Metabolism

Iron is essential in the catalysis of many, if not most, enzymatic reactions that involve electron transfer and play a critical role in cellular survival. However, free iron is toxic due to its ability to generate free radicals via the Fenton reaction and to catalyze lipid peroxidation chain reactions [83,84]. Thus, the control of this necessary but potentially toxic metal is important for human health and disease. Iron homeostasis is tightly controlled by the regulation of its cellular import, storage, and intracellular movement [85,86].

### 4.1. Cellular Iron Metabolism

In animal cells, non-heme iron is transported into cells through two main pathways: transferrin (Tf)-bound iron uptake and non-Tf-bound iron (NTBI) uptake. NTBI uptake occurs when the body absorbs dietary iron from the intestinal lumen, or when Tf becomes saturated with iron because of iron overload. Although the exact NTBI uptake pathway is unclear, it is proposed that reductases, such as duodenal cytochrome *b* (Dcytb), reduce Fe^3+^ to Fe^2+^, which is then imported into cells via divalent metal transporter 1 (DMT1) or ZRT/IRT-like proteins (ZIPs) [87–89].

Under physiological conditions, almost all serum iron is bound to Tf. The uptake of Tf-bound iron through Tf receptor-1 (TfR1) is the major pathway for the delivery of iron into cells [85,86]. Tf-dependent iron delivery begins with the binding of diferric Tf to TfR1 on the cell surface, followed by the endocytosis of the Tf-TfR1 complex. As pH decreases during endosome maturation and fusion with lysosomes, Fe^3+^ dissociates from Tf, and both Tf and TfR1 recycle to the cell surface for another round of iron uptake. A ferrireductase (Steap3) then reduces dissociated Fe^3+^ to Fe^2+^ within the endosomal/lysosomal compartment. Fe^2+^ subsequently exits the endosomal/lysosomal compartment into the cytosol via DMT1 or ZIP14 [90,91]. The release of Fe^2+^ from endosomal/lysosomal membranes appears to involve an Fe^2+^/H^+^ exchange mechanism [92]. Iron released to the cytosol is in a soluble, chelatable state, which constitutes the labile iron pool (LIP). From this pool, iron can be stored in ferritin, utilized for metabolism (*e.g.*, imported into mitochondria for the synthesis of heme and Fe-S clusters), used to generate ROS, or exported from the cell by ferroportin 1 (FPN1) [85,86]. Notably, lysosomes are additionally involved in intracellular iron recycling because of the degradation of many macromolecules containing iron inside the lysosomal lumen [93].

### 4.2. Mitochondrial Iron Metabolism

Mitochondria utilize iron for the synthesis of heme and Fe-S clusters [94–97]. Iron moves into mitochondria using the following hypothesized mechanisms: (i) Iron-loaded endosomes/lysosomes interact directly with mitochondria by a “kiss-and-run” mechanism, leading to mitochondrial iron uptake [98]. (ii) Iron from ferritin transfers into mitochondria after ferritin complex degradation [99–101]. These mechanisms remain incompletely understood and need further study.

Two transporters, the mitochondrial calcium uniporter (MCU) and the two isoforms of mitoferrin (Mfrn1/2), play essential roles in transporting iron across the inner membrane. MCU catalyzes the electrogenic mitochondrial uptake of both Ca^2+^ and Fe^2+^ driven by the negative inside mitochondrial ΔΨ, which is blocked by the specific MCU inhibitor, Ru360 [81,102–104]. Mfrn1 and its paralog Mfrn2 also mediate mitochondrial iron uptake in erythroid and non-erythroid cells, respectively [105,106]. Because mitochondrial iron uptake is needed for heme synthesis, the deletion of Mfrn1 in hematopoietic tissues leads to anemia [106]. Some evidence indicates that Mfrn2 physically interacts with MCU, possibly as a component and/or regulator of the MCU complex [107].

Once imported into mitochondria, iron is utilized for the synthesis of heme and Fe-S clusters, which are incorporated into respiratory and other enzymes inside the mitochondria or exported to the cytosol to become prosthetic groups for cytosolic enzymes. Mitochondrial iron is also stored in mitochondrial ferritin (FTMT) [108].

### 4.3. Role of Iron in Common Models of Acute Liver Injury

However, when mitochondrial iron uptake results in iron overload and simultaneously H_2_O_2_ is generated by mitochondrial respiration that cannot be detoxified by antioxidant systems, Fe^2+^ and H_2_O_2_ react to form •OH, leading to lipid peroxidation, mitochondrial dysfunction, DNA damage, and a form of necrotic cell death now called ferroptosis [83,104,109,110]. Iron chelators like desferal and starch-desferal decrease mitochondrial ROS formation, MPT opening, and cell killing in cultured rat hepatocyte models of hypoxia/ischemia [104]. Desferal also protects against lethal injury to cultured hepatocytes from *tert*-butyl hydroperoxide, as does the lipid radical scavenger, N,N-diphenyl-p-phenylenediamine (DPPD) [111,112]. Another iron chelator, deferasirox, protects against concanavalin A-induced hepatic injury and fibrosis in rats [113]. Cytoprotection by iron chelators against hypoxia/ischemia, oxidative stress, and APAP hepatotoxicity infers a critical role for iron in the pathogenesis of injury, most likely by catalyzing •OH formation and subsequent lipid peroxidation [104,111,114–117].

## 5. Iron and Acetaminophen Hepatotoxicity

### 5.1. Evidence for Mitochondrial Iron Uptake in Acetaminophen Hepatotoxicity

After APAP overdose, the mitochondrial generation of ROS is a critical factor triggering the MPT, and iron promotes this oxidative stress [81]. Iron chelators and antioxidants that scavenge ROS protect against APAP toxicity in vitro and in vivo [114,118–121]. Treatment with the iron chelator, desferal (also called deferoxamine or desferrioxamine), increases the time required for APAP to induce ROS and mitochondrial dysfunction in cultured mouse hepatocytes [122]. After iron chelation with desferal, the addition of iron to the culture medium restores the sensitivity of hepatocytes to APAP toxicity in vitro [114,120]. Moreover, the treatment of mouse hepatocytes with the iron donor 3,5,5-trimethyl-hexanoyl ferrocene (TMHF) causes APAP-induced ROS formation and mitochondrial dysfunction to occur at earlier time points than APAP treatment alone, which is partially prevented by desferal [122].

Several fluorescent probes can visualize intracellular iron movement between organelles. The exogenously added calcein-acetoxymethylester (AM) is de-esterified in the cytosol to release calcein-free acid, whose fluorescence is quenched by chelatable Fe^2+^ [92,104,123]. Mitoferrofluor (MFF) is another iron indicator that accumulates electrophoretically into mitochondria in response to ΔΨ and then binds covalently to mitochondrial proteins. Like green-fluorescing calcein, red-fluorescing MFF is quenched by chelatable Fe^2+^ [124]. Calcein and MFF can be used together or in combination with fluorescent indicators of mitochondrial ΔΨ, such as red-fluorescing tetramethylrhodamine methylester (TMRM) and green-fluorescing rhodamine 123 (Rh123) [81,124]. To visualize lysosomes, cells can be pre-loaded with red-fluorescing rhodamine-dextran, which is taken up via endocytosis and delivered to the lysosomes [116].

**Figure 1.**
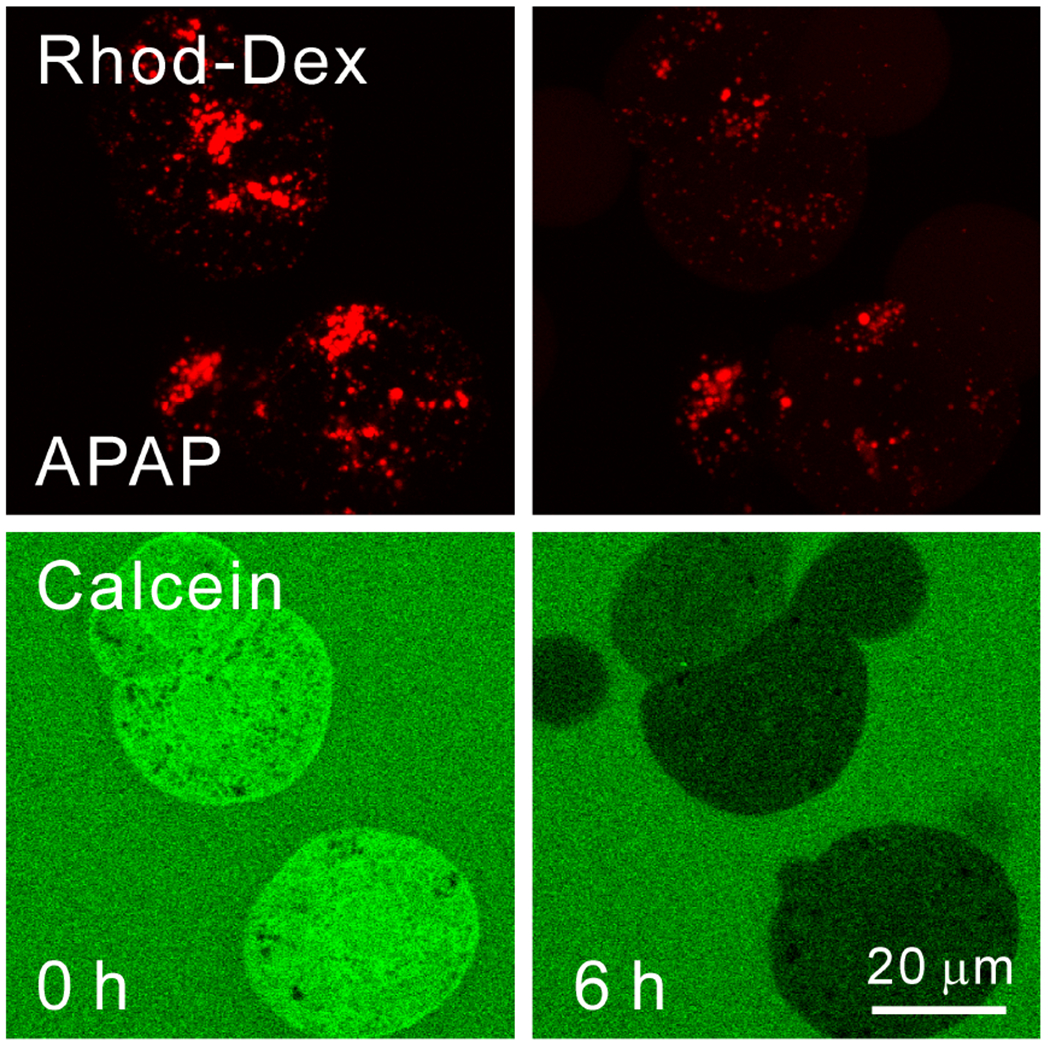
Acetaminophen-dependent lysosomal permeabilization and release of Fe^2+^ into the cytosol. Wildtype mouse hepatocytes were isolated from mice injected with 70 kDa rhodamine-dextran and then loaded with 1 μM calcein-AM. Rhodamine-dextran labeled lysosomes, whereas calcein-AM was de-esterified to release calcein-free acid into the cytosol. In the presence of 20 mM of fructose plus 5 mM of glycine to prevent cell death after APAP-induced disruption of mitochondrial metabolism, hepatocytes were then exposed to acetaminophen (APAP, 10 mM). Before APAP (0 h), rhodamine-dextran-labeled lysosomes were intact, and cytosolic calcein fluorescence was bright in comparison to the fluorescence of 300 μM of calcein-free acid placed in the extracelluar medium. At 4 h after APAP, many rhodamine-dextran-labeled lysosomes disappeared in parallel with the quenching of calcein fluorescence. This calcein quenching signified increased cytosolic chelatable Fe^2+^. As lysosomes disappeared, diffuse red fluorescence appeared in the cytosol, signifying that acetaminophen permeabilized many lysosomes. After [116].

In cultured mouse hepatocytes, APAP causes lysosomes to rupture and release rhodamine-dextran into the cytosol within 4 h (Figure 1, top row). The mechanism underlying APAP-induced lysosomal rupture is not known. The APAP metabolite, NAPQI, may react covalently with lysosomal membrane components to cause the rupture. In parallel, cytosolic calcein fluorescence becomes quenched, though this is not the case for the fluorescence of calcein-free acid added to the extracellular medium, indicating an increase in cytosolic Fe^2+^ due to its release from lysosomes (Figure 1, bottom row) [81]. Starch-desferal suppresses the increase in cytosolic and mitochondrial Fe^2+^ after APAP [81]. Since membrane-impermeant starch-desferal is taken up via endocytosis into the lysosomal/endosomal compartment like rhodamine-dextran, the prevention of APAP-induced increases in cytosolic and mitochondrial Fe^2+^ by starch-desferal confirms that endosomes/lysosomes are the source of mobilizable chelatable iron entering the cytosol and mitochondria during APAP hepatotoxicity. Other sources of iron may promote the Fenton reaction in mitochondria. For example, ROS promote heme oxygenase 1 (HO-1) translocation to mitochondria in cardiomyocytes, leading to iron release from heme [125]. Further study is needed to determine whether HO-1 is involved in APAP hepatotoxicity.

### 5.2. Role of the Mitochondrial Calcium Uniporter in Mitochondrial Iron Uptake during Acetaminophen Hepatotoxicity

**Figure 2.**
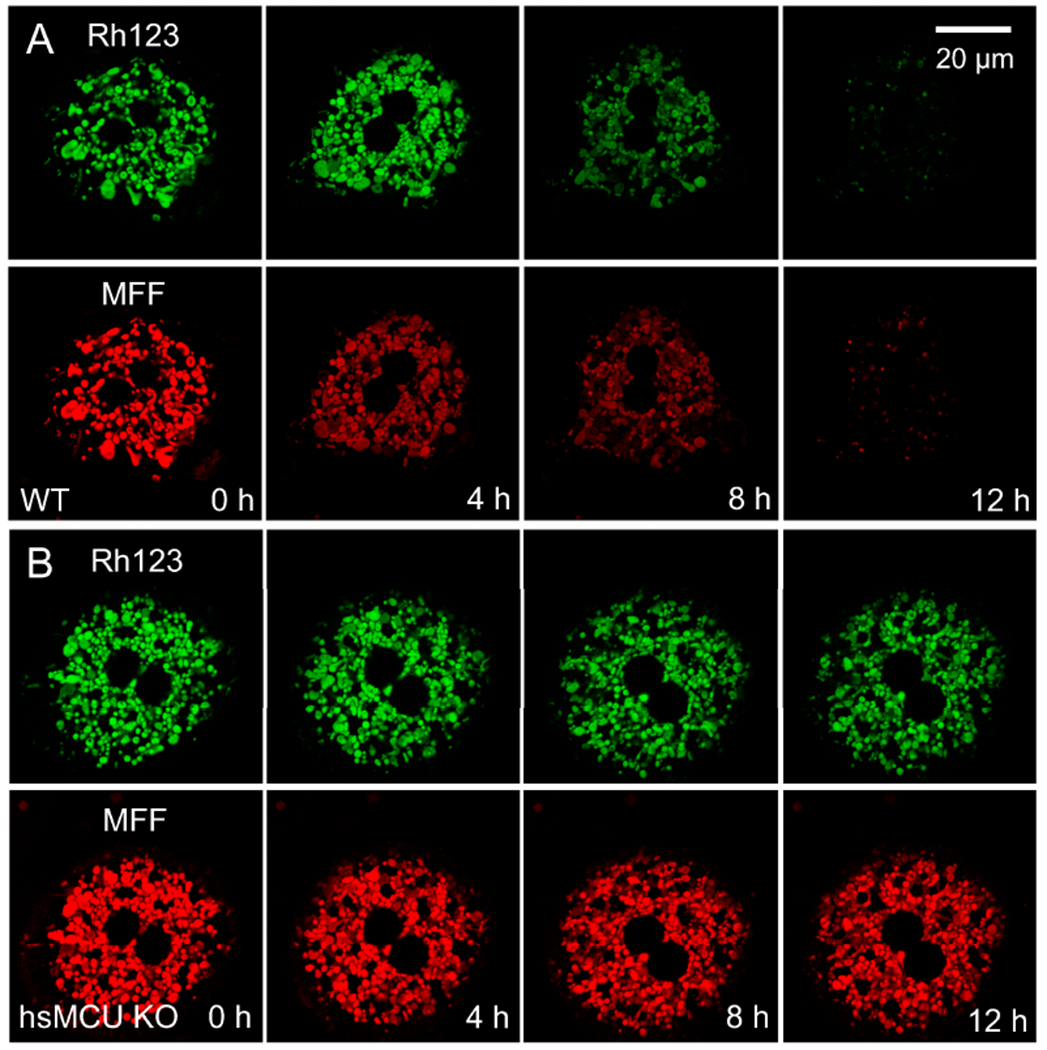
Suppression of mitochondrial iron uptake and depolarization after acetaminophen treatment of hepatocytes deficient in the mitochondrial calcium uniporter. Wildtype and hsMCU KO hepatocytes were loaded with 300 nM of Rh123 plus 1 μM of MFF and exposed to 10 mM APAP in the presence of 20 mM of fructose plus 5 mM of glycine. Rh123 is a green-fluorescing indicator of mitochondrial ΔΨ. Mitoferrofluor (MFF) accumulates electrophoretically into mitochondria, binds covalently, and becomes quenched as mitochondrial Fe^2+^ increases. (**A**) In wildtype (WT) hepatocytes, red mitochondrial MFF fluorescence was bright at 0 h but subsequently quenched progressively, beginning within 4 h and becoming virtually complete after 12 h (bottom row). Mitochondrial depolarization (loss of green Rh123 fluorescence) began to occur at 8 h and was complete after 12 h (top row). (**B**) In hsMCU KO hepatocytes, mitochondrial MFF quenching and mitochondrial depolarization were suppressed after APAP. After [126].

**Figure 3.**
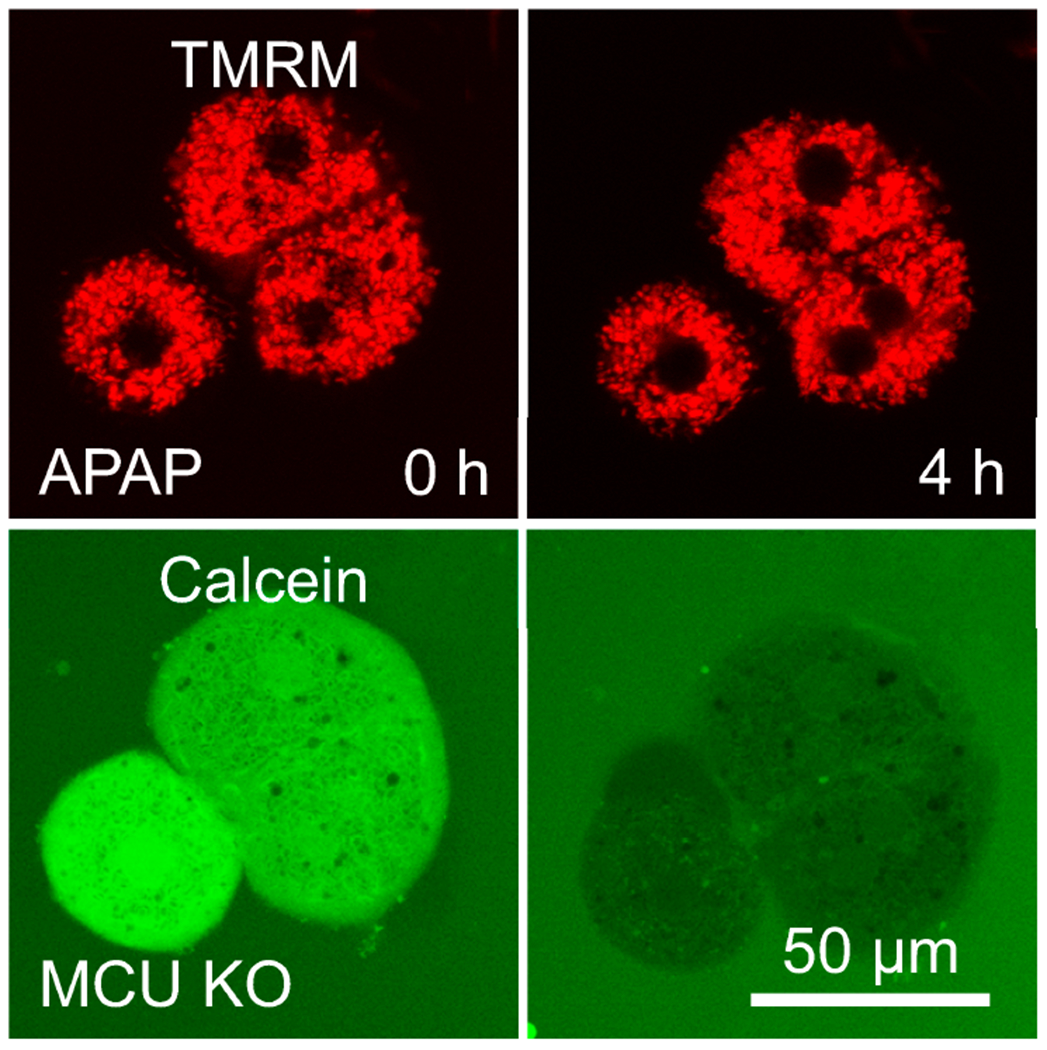
Increased cytosolic Fe^2+^ in MCU-deficient hepatocytes after acetaminophen. Hepatocytes were loaded with 300 nM of TMRM plus 1 μM of calcein-AM and incubated with 300 μM of calcein-free before exposure to 10 mM APAP in the presence of 20 mM fructose plus 5 mM glycine. TMRM is a red-fluorescing indicator of mitochondrial ΔΨ. When MCU-deficient hepatocytes were exposed to 10 mM APAP, mitochondrial depolarization (loss of TMRM fluorescence) was suppressed. However, the green cytosolic calcein fluorescence decreased substantially similarly to wildtype hepatocytes, signifying increased cytosolic chelatable Fe^2+^. After [126].

Movement into the mitochondria of Fe^2+^ released from ruptured lysosomes is mediated by MCU, an electrogenic Ca^2+^ transporter that also conducts Fe^2+^, since the MCU inhibitors, Ru360 and minocycline, block MFF quenching but not calcein quenching after APAP [81]. Further support for this role of MCU is provided by studies using mice with a hepatocyte-specific MCU (hsMCU) deficiency. In wildtype hepatocytes, mitochondrial MFF fluorescence is bright but subsequently progressively decreases after APAP exposure, beginning within 4 h and becoming virtually complete after 12 h (Figure 2A, bottom row). In parallel, mitochondrial depolarization (the loss of Rh123 fluorescence), signifying the onset of the MPT, begins to occur within 8 h and is complete within 12 h (Figure 2A, top row). By contrast, in hsMCU KO hepatocytes that are deficient in MCU, mitochondrial MFF quenching and mitochondrial depolarization are suppressed after APAP (Figure 2B). Nonetheless, cytosolic calcein fluorescence is just as strongly quenched after APAP in MCU-deficient hepatocytes as in wildtype hepatocytes showing that lysosomes still release Fe^2+^ (Figures 1 and 3). Both in vitro and in vivo, lysosomal iron chelation with starch-desferal and the inhibition of MCU-mediated mitochondrial iron uptake protect against APAP-induced hepatotoxicity [81,116,117,126]. Notably, both the global- and hepatocyte-specific deficiency of MCU decreases APAP hepatotoxicity in vivo as assessed by ALT release and necrosis by histology without altering hepatic APAP metabolism [126]. In addition, the co-treatment of APAP with FeSO_4_ dramatically increases APAP-induced hepatotoxicity, which is prevented by desferal [27].

### 5.3. Possible Roles of Kupffer Cells and JNK in Iron-Dependency of Acetaminophen Hepatotoxicity

Kupffer cells are liver-resident macrophages that are involved in the phagocytosis of senescent red blood cells and the recycling of iron [127]. Kupffer cells are also a potential source of oxidant stress promoting cell death [128]. Human and mouse studies indicate that Kupffer cells and infiltrating monocyte-derived macrophages have both injury-promoting and injury-repair functions after APAP overdose [129–133]. Although MCU deficiency in hepatocytes decreases liver necrosis and ALT release after APAP in mice, MCU deficiency in Kupffer cells does not alter APAP hepatotoxicity [126].

JNK activation in the cytosol and translocation of p-JNK to mitochondria are important early events promoting the MPT and cell death in APAP hepatotoxicity [32]. Recent in vivo studies in mice show that neither desferal nor Fe^2+^ treatment affects JNK activation and its translocation to mitochondria after APAP overdose [27]. These findings suggest that the effect of iron is not at the early stages of the response to APAP but specifically at later events within mitochondria.

### 5.4. “Two Hit” Hypothesis

**Figure 4.**
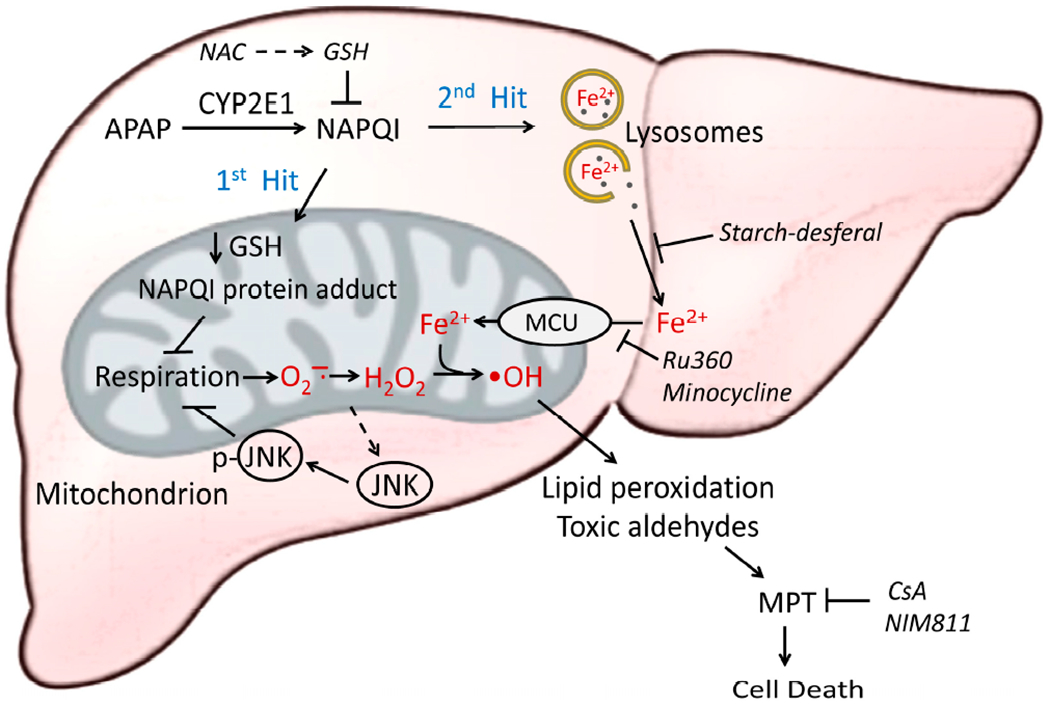
Two-hit model of APAP hepatotoxicity. After an overdose of APAP, the first hit occurs when APAP causes GSH depletion, NAPQI protein adduct formation, and the inhibition of mitochondrial respiration, which induces O_2_^•−^ and H_2_O_2_ formation. ROS-induced JNK phosphorylation and activation further enhance respiratory inhibition and mitochondrial ROS formation. The second hit occurs when NAPQI damages lysosomes and releases Fe^2+^ into the cytosol, which is then taken up into mitochondria via the electrogenic MCU to promote intramitochondrial •OH formation by the Fenton reaction. •OH, in turn, induces lipid peroxidation, the formation of toxic aldehydes, MPT onset, and mitochondrial bioenergetic failure, leading to the loss of cell viability. Starch-desferal chelates lysosomal iron to prevent the release of chelatable iron after lysosomal disruption and subsequent uptake into mitochondria to promote •OH formation. Ru360 and minocycline block mitochondrial iron uptake via MCU to also suppress iron-catalyzed •OH formation in the mitochondrial matrix. CsA and NIM811 inhibit MPT. Blocking either hit protects against APAP-induced hepatic injury.

Overall, these results support a “two hit” hypothesis for the role of oxidative stress and iron in APAP hepatotoxicity (Figure 4) [81] (see also [104]). In the first hit, CYP2E1 metabolizes APAP to NAPQI, which induces mitochondrial protein adduct formation, the disruption of mitochondrial respiration, and consequent generation of (O_2_^•−^ and H_2_O_2_. These ROS also activate JNK, which translocates to mitochondria to further inhibit respiration with the feed-forward effect of enhancing mitochondrial ROS generation even more. In the second hit, toxic NAPQI causes lysosomal breakdown and the release of chelatable Fe^2+^ into the cytosol. Fe^2+^ is then taken up into mitochondria via MCU. In the presence of O_2_^•−^ and H_2_O_2_, such mitochondrial Fe^2+^ loading induces •OH formation via the Fenton reaction, which in turn causes MPT onset, mitochondria depolarization, bioenergetic failure, and cell death. Iron imported into mitochondria also facilitates protein nitration by peroxynitrite (ONOO^−^), which is formed from the reaction of O_2_^•−^ with nitric oxide (NO) [27].

### 5.5. Ferroptosis during Acetaminophen Hepatotoxicity

Iron has long been known to promote lipid peroxidation and cell death in various models of cell injury (see [112,115,120,134,135]). During APAP toxicity to cultured hepatocytes, DPPD, a scavenger of lipid radicals, prevents both lipid peroxidation and cell death [111,136]. Similarly, ferrostatin-1, a scavenger of alkoxyl radicals that propagate lipid peroxidation chain reactions, protects against APAP-induced hepatotoxicity in mice [137]. Non-apoptotic iron-dependent cell death involving lipid peroxidation and mitochondrial iron-loading has more recently been named ferroptosis [110,138]. A novel ferroptosis inhibitor, mifepristone, prevents APAP-induced hepatotoxicity in vitro and in mice in vivo [139], and growth arrest-specific 1 (GAS1) overexpression promotes ferroptosis and aggravates APAP-induced hepatocellular injury both in vitro and in vivo [140].

### 5.6. Role of Peroxynitrite and Protein Nitration in Acetaminophen Hepatotoxicity

**Figure 5.**
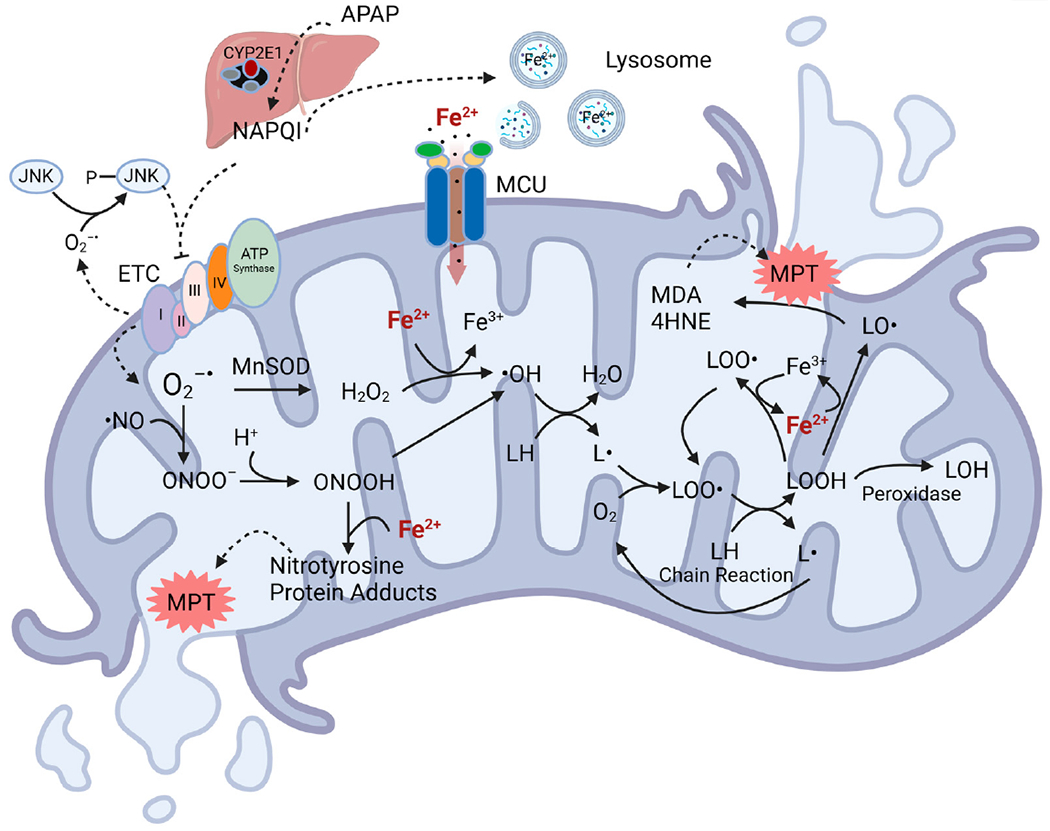
Role of iron in oxidative stress in APAP-induced mitochondrial damage. After an overdose of APAP, NAPQI binds to mitochondrial proteins to inhibit mitochondrial respiration. Respiratory inhibition leads to increased levels of flavin semiquinones and ubisemiquinone, which react with oxygen to form O_2_•^−^. Such respiratory inhibition and ROS generation are further amplified through ROS-driven JNK activation. O_2_•^−^ reacts rapidly with NO to form ONOO^−^. The iron influx into mitochondria facilitates the reaction of ONOO^−^ with proteins to produce nitrotyrosine adducts, ultimately promoting the MPT. SOD2 in mitochondria also converts O_2_•^−^ to H_2_O_2_. Fe^2+^, which is released from damaged lysosomes, is taken up into mitochondria via MCU and reacts with H_2_O_2_ to form the toxic •OH, which induces L• formation. L• then initiates an oxygen-dependent chain reaction generating peroxyl radicals (LOO•) and lipid peroxides (LOOHs). In the presence of Fe^2+^, LOOH produces LO•. The beta scission of LO• then leads to the formation of reactive aldehydes like MDA and 4HNE, which also promote MPT onset. This figure was created with BioRender.com.

Protein nitration is an important pathophysiological event in APAP hepatotoxicity [141,142]. During APAP overdose, respiratory chain dysfunction leads to the generation of O_2_•^−^, which reacts with NO to form reactive and toxic ONOO^−^ in the mitochondrial matrix [27,143]. The mitochondrial uptake of iron released from lysosomes then promotes ONOO^−^-dependent nitration of protein tyrosine residues to form nitrotyrosine protein adducts [27,144]. This stress further induces the MPT in APAP toxicity (Figure 5). Consistent with this mechanism in vivo after APAP overdose, desferal and the MCU blocker, minocycline, attenuate immunostaining for nitrotyrosine protein adducts and the release of the mitochondrial intermembrane protein, cytochrome *c*, which is a consequence of mitochondrial swelling after MPT onset [27]. The co-treatment of APAP with FeSO_2_ in mice further increases nitrotyrosine staining and the release of cytochrome *c*, as well as causing lipid peroxidation, which desferal inhibits [27]. Moreover, the mitochondria-specific SOD mimetic, mito-TEMPO, protects against APAP-induced liver injury and nitrotyrosine protein adduct formation in mice [145].

### 5.7. Aldehydes as Drivers of Acetaminophen Hepatotoxicity

•OH from Fenton chemistry reacts with unsaturated lipids to initiate a lipid peroxidation chain reaction with the formation of lipid radicals (L•), lipid peroxides (LOOH), and peroxyl radicals (LOO•). Iron is an important catalyst to then promote a subsequent alkoxyl radical (LO•) and more LOO• formation. Notably, the spontaneous non-enzymatic beta-scission of LO• generates a variety of aldehydes, including malondialdehyde (MDA) and 4-hydroxynonenal (4-HNE), which are often used as biomarkers for lipid peroxidation. However, MDA, 4-HNE, and other aldehydes formed downstream of lipid peroxidation are toxic, reactive, and mutagenic, with MDA reported to be the most mutagenic and 4-HNE the most toxic [146–148].

Lipid peroxidation in APAP hepatotoxicity was initially indicated by the appearance of exhalated hydrocarbons in mice in vivo and by MDA formation in liver homogenates in vitro that inducers and inhibitors of P450 enzymes, respectively, up and down modulate [149,150]. However, these studies were performed with mice fed a vitamin E-deficient diet high in polyunsaturated fatty acids that made the animals sensitive to lipid peroxidation induced by APAP [150,151]. A follow-up study with mice fed a regular diet showed minimal evidence for lipid peroxidation after APAP [152]. Furthermore, mice fed a diet high in vitamin E diet do not show decreased APAP hepatotoxicity, suggesting that endogenous defense mechanisms are normally sufficient to prevent excessive lipid peroxidation after APAP [152]. Additionally, the co-treatment of Fe^2+^ with APAP increases lipid peroxidation in vivo in mice, which desferal almost completely prevents [27,153]. Nonetheless, other reports show that APAP stimulates lipid peroxidation in isolated mouse and rat hepatocytes in vitro [154,155], and mass spectroscopy reveals lipid peroxides derived from n-6 fatty acids, mainly from arachidonic acid, after APAP overdose [137]. Moreover, 4-HNE adduct formation increases after APAP in mice fed normal chow [156].

N-(1,3-benzodioxol-5-ylmethyl)-2,6-dichlorobenzamide (Alda-1) is an activator of mitochondrial aldehyde dehydrogenase-2 (ALDH2) and is responsible for detoxifying aldehyde oxidation to fatty acids [157]. After APAP in vivo, Alda-1 decreases 4-HNE adduct formation, APAP-induced liver injury, and mitochondrial dysfunction, indicating that lipid peroxidation-derived aldehydes are important mediators of APAP hepatotoxicity. Lipid peroxidation may occur relatively selectively in mitochondria that are the source of •OH from Fenton chemistry and whose membranes are enriched in arachidonic acid.

## 6. Summary and Conclusions

Iron-catalyzed free radical generation in mitochondria plays an important role in APAP toxicity (Figure 5). Initially, the toxic APAP metabolite, NAPQI, binds to mitochondrial proteins to inhibit mitochondrial respiration. Inhibited respiration leads to increased levels of ubisemiquinone and flavin semiquinone, which transfer their unpaired electrons to oxygen to form O_2_•^−^. Respiratory inhibition is further amplified through JNK activation, leading to greater O_2_•^−^ generation. O_2_•^−^ reacts with nitric oxide to produce peroxynitrite or is converted to H_2_O_2_ by SOD. Since NAPQI depletes GSH after APAP overdose, GSH is no longer available to detoxify peroxynitrite and H_2_O_2_, as would occur normally. NAPQI also damages lysosomes, causing Fe^2+^ release into the cytosol and subsequent uptake into mitochondria via the MCU. Mitochondrial loading with Fe^2+^ facilitates nitrotyrosine protein adduct formation and Fenton chemistry with H_2_O_2_ to produce the highly reactive •OH. •OH, in turn, causes lipid peroxidation, the formation of toxic aldehydes, and induction of the MPT, ultimately leading to cell death. Accordingly, blocking pathways of iron movement into mitochondria via MCU, preventing iron-related mitochondrial •OH and ONOO^−^ formation, and accelerating aldehyde metabolism are potential novel strategies to intervene against APAP hepatotoxicity in a clinical setting.

## Data Availability

No new data were created or analyzed. Data sharing is not applicable to this article.

## Abbreviations

•OH: hydroxyl radical
ΔΨ: membrane potential
Alda-1: N-(1,3-benzodioxol-5-ylmethyl)-2,6-dichlorobenzamide
ALDH2: mitochondrial aldehyde dehydrogenase-2
ALF: acute liver failure
AM: acetoxymethylester
ANT: adenine nucleotide translocator
APAP: N-acetyl-para-aminophenol, acetaminophen
CsA: cyclosporin A
CypD: cyclophilin D
DMT1: divalent metal transporter 1
DPD: dipyridyl
DPPD’ N: N’-diphenyl-p-phenylenediamine
FPN1: ferroportin 1
FTMT: mitochondrial ferritin
GAS1: growth arrest-specific 1
GSH: glutathione
HDM: hormonally defined medium
4-HNE: 4-hydroxynonenal
HO-1: heme oxygenase 1
JNK: c-Jun N-terminal protein kinase
LIP: labile iron pool
L•: lipid radicals
LOO•: peroxyl radical
LOOH: lipid peroxide
MAPK: mitogen-activated protein kinase
MASLD: metabolic dysfunction-associated steatotic liver disease
MCU: mitochondrial calcium uniporter
MDA: malondialdehyde
MFF: mitoferrofluor
Mfrn: mitoferrin
MPT: mitochondrial permeability transition
NAC: N-acetylcysteine
NAPQI: N-acetyl-p-benzoquinone imine
NO: nitric oxide
NTBI: non- transferrin-bound iron
ONOO^−^: peroxynitrite
p-JNK: phospho-JNK
PI: propidium iodide
PT: permeability transition
PTPN6: protein tyrosine phosphatase nonreceptor type 6
Rh123: rhodamine 123
ROS: reactive oxygen species
SAB: SH3 domain-binding protein that preferentially associates with Bruton’s tyrosine kinase
SOD: superoxide dismutase
Tf: transferrin
TfR1: Tf receptor-1
TMHF: 3,5,5-trimethyl-hexanoyl ferrocene
TMRM: tetramethylrhodamine methylester
VDAC: voltage-dependent anion channels
ZIPs: ZRT/IRT-like proteins

## References

[1] Fisher ES; Curry SC Evaluation and treatment of acetaminophen toxicity. Adv. Pharmacol 2019, 85, 263–272. 31307590 10.1016/bs.apha.2018.12.004

[2] Michna E; Duh MS; Korves C; Dahl JL Removal of opioid/acetaminophen combination prescription pain medications: Assessing the evidence for hepatotoxicity and consequences of removal of these medications. Pain Med. 2010, 11, 369–378. 20447306 10.1111/j.1526-4637.2010.00811.x

[3] Blieden M; Paramore LC; Shah D; Ben-Joseph R. A perspective on the epidemiology of acetaminophen exposure and toxicity in the United States. Expert Rev. Clin. Pharmacol 2014, 7, 341–348. 24678654 10.1586/17512433.2014.904744

[4] Mitchell JR; Jollow DJ; Potter WZ; Gillette JR; Brodie BB Acetaminophen-induced hepatic necrosis. IV. Protective role of glutathione. J. Pharmacol. Exp. Ther 1973, 187, 211–217. 4746329

[5] McGill MR; Jaeschke H Metabolism and disposition of acetaminophen: Recent advances in relation to hepatotoxicity and diagnosis. Pharm. Res 2013, 30, 2174–2187. 23462933 10.1007/s11095-013-1007-6 PMC3709007

[6] Ramachandran A; Jaeschke H. Mitochondria in Acetaminophen-Induced Liver Injury and Recovery: A Concise Review. Livers 2023, 3, 219–231. 37377765 10.3390/livers3020014 PMC10299745

[7] Goyal RK; Rajan SS; Essien EJ; Sansgiry SS Effectiveness of FDA’s new over-the-counter acetaminophen warning label in improving consumer risk perception of liver damage. J. Clin. Pharm. Ther 2012, 37, 681–685. 22958105 10.1111/j.1365-2710.2012.01371.x

[8] Schilling A; Corey R; Leonard M; Eghtesad B. Acetaminophen: Old drug, new warnings. Cleve Clin. J. Med 2010, 77, 19–27. 20048026 10.3949/ccjm.77a.09084

[9] McGill MR; James LP; McCullough SS; Moran JH; Mathews SE; Peterson EC; Fleming DP; Tripod ME; Vazquez JH; Kennon-McGill S; Short-Term Safety of Repeated Acetaminophen Use in Patients with Compensated Cirrhosis. Hepatol. Commun 2022, 6, 361–373. 34558847 10.1002/hep4.1810 PMC8793989

[10] Myers RP; Shaheen AA; Li B; Dean S; Quan H. Impact of liver disease, alcohol abuse, and unintentional ingestions on the outcomes of acetaminophen overdose. Clin. Gastroenterol. Hepatol 2008, 6, 918–925, quiz 837. 18486561 10.1016/j.cgh.2008.02.053

[11] Bacle A; Pronier C; Gilardi H; Polard E; Potin S; Scailteux LM Hepatotoxicity risk factors and acetaminophen dose adjustment, do prescribers give this issue adequate consideration? A French university hospital study. Eur. J. Clin. Pharmacol 2019, 75, 1143–1151. 30972451 10.1007/s00228-019-02674-5

[12] Vogt BL; Richie JP Jr. Fasting-induced depletion of glutathione in the aging mouse. Biochem. Pharmacol 1993, 46, 257–263. 8347148 10.1016/0006-2952(93)90412-p

[13] Kurtovic J; Riordan SM Paracetamol-induced hepatotoxicity at recommended dosage. J. Intern. Med 2003, 253, 240–243. 12542566 10.1046/j.1365-2796.2003.01097.x

[14] Zimmerman HJ; Maddrey WC Acetaminophen (paracetamol) hepatotoxicity with regular intake of alcohol: Analysis of instances of therapeutic misadventure. Hepatology 1995, 22, 767–773. 7657281

[15] James LP; Alonso EM; Hynan LS; Hinson JA; Davern TJ; Lee WM; Squires RH; Pediatric Acute Liver Failure Study, G. Detection of acetaminophen protein adducts in children with acute liver failure of indeterminate cause. Pediatrics 2006, 118, e676–e681. 16950959 10.1542/peds.2006-0069

[16] Chidiac AS; Buckley NA; Noghrehchi F; Cairns R. Paracetamol (acetaminophen) overdose and hepatotoxicity: Mechanism, treatment, prevention measures, and estimates of burden of disease. Expert Opin. Drug Metab. Toxicol 2023, 19, 297–317. 37436926 10.1080/17425255.2023.2223959

[17] Suzuki A; Yuen N; Walsh J; Papay J; Hunt CM; Diehl AM Co-medications that modulate liver injury and repair influence clinical outcome of acetaminophen-associated liver injury. Clin. Gastroenterol. Hepatol 2009, 7, 882–888. 19362607 10.1016/j.cgh.2009.03.034

[18] Rinella ME; Lazarus JV; Ratziu V; Francque SM; Sanyal AJ; Kanwal F; Romero D; Abdelmalek MF; Anstee QM; Arab JP; A multisociety Delphi consensus statement on new fatty liver disease nomenclature. Hepatology 2023, 78, 1966–1986. 37363821 10.1097/HEP.0000000000000520 PMC10653297

[19] Michaut A; Moreau C; Robin MA; Fromenty B Acetaminophen-induced liver injury in obesity and nonalcoholic fatty liver disease. Liver Int. 2014, 34, e171–e179. 24575957 10.1111/liv.12514

[20] Larson AM Acetaminophen hepatotoxicity. Clin. Liver Dis 2007, 11, 525–548. 17723918 10.1016/j.cld.2007.06.006

[21] Bunchorntavakul C; Reddy KR Acetaminophen-related hepatotoxicity. Clin. Liver Dis 2013, 17, 587–607. 24099020 10.1016/j.cld.2013.07.005

[22] Rumack BH; Peterson RC; Koch GG; Amara IA Acetaminophen overdose. 662 cases with evaluation of oral acetylcysteine treatment. Arch. Intern. Med 1981, 141, 380–385. 7469629 10.1001/archinte.141.3.380

[23] Perry HE; Shannon MW Efficacy of oral versus intravenous N-acetylcysteine in acetaminophen overdose: Results of an open-label, clinical trial. J. Pediatr 1998, 132, 149–152. 9470017 10.1016/s0022-3476(98)70501-3

[24] Yeates PJ; Thomas SH Effectiveness of delayed activated charcoal administration in simulated paracetamol (acetaminophen) overdose. Br. J. Clin. Pharmacol 2000, 49, 11–14. 10606832 10.1046/j.1365-2125.2000.00107.x PMC2014891

[25] Russo MW; Galanko JA; Shrestha R; Fried MW; Watkins P. Liver transplantation for acute liver failure from drug induced liver injury in the United States. Liver Transpl. 2004, 10, 1018–1023. 15390328 10.1002/lt.20204

[26] Athersuch TJ; Antoine DJ; Boobis AR; Coen M; Daly AK; Possamai L; Nicholson JK; Wilson ID Paracetamol metabolism, hepatotoxicity, biomarkers and therapeutic interventions: A perspective. Toxicol. Res 2018, 7, 347–357. 10.1039/c7tx00340d PMC6062253 30090586

[27] Adelusi OB; Ramachandran A; Lemasters JJ; Jaeschke H. The role of Iron in lipid peroxidation and protein nitration during acetaminophen-induced liver injury in mice. Toxicol. Appl. Pharmacol 2022, 445, 116043. 35513057 10.1016/j.taap.2022.116043 PMC9843742

[28] Anandatheerthavarada HK; Addya S; Dwivedi RS; Biswas G; Mullick J; Avadhani NG Localization of multiple forms of inducible cytochromes P450 in rat liver mitochondria: Immunological characteristics and patterns of xenobiotic substrate metabolism. Arch. Biochem. Biophys 1997, 339, 136–150. 9056243 10.1006/abbi.1996.9855

[29] Robin MA; Anandatheerthavarada HK; Fang JK; Cudic M; Otvos L; Avadhani NG Mitochondrial targeted cytochrome P450 2E1 (P450 MT5) contains an intact N terminus and requires mitochondrial specific electron transfer proteins for activity. J. Biol. Chem 2001, 276, 24680–24689. 11325963 10.1074/jbc.M100363200

[30] Massart J; Begriche K; Hartman JH; Fromenty B. Role of Mitochondrial Cytochrome P450 2E1 in Healthy and Diseased Liver. Cells 2022, 11, 288. 35053404 10.3390/cells11020288 PMC8774478

[31] Kon K; Kim JS; Jaeschke H; Lemasters JJ Mitochondrial permeability transition in acetaminophen-induced necrosis and apoptosis of cultured mouse hepatocytes. Hepatology 2004, 40, 1170–1179. 15486922 10.1002/hep.20437

[32] Hanawa N; Shinohara M; Saberi B; Gaarde WA; Han D; Kaplowitz N. Role of JNK translocation to mitochondria leading to inhibition of mitochondria bioenergetics in acetaminophen-induced liver injury. J. Biol. Chem 2008, 283, 13565–13577. 18337250 10.1074/jbc.M708916200 PMC2376214

[33] Hu J; Ramshesh VK; McGill MR; Jaeschke H; Lemasters JJ Low Dose Acetaminophen Induces Reversible Mitochondrial Dysfunction Associated with Transient c-Jun N-Terminal Kinase Activation in Mouse Liver. Toxicol. Sci 2016, 150, 204–215. 26721299 10.1093/toxsci/kfv319 PMC5009618

[34] Dunn KW; Martinez MM; Wang Z; Mang HE; Clendenon SG; Sluka JP; Glazier JA; Klaunig JE Mitochondrial depolarization and repolarization in the early stages of acetaminophen hepatotoxicity in mice. Toxicology 2020, 439, 152464. 32315716 10.1016/j.tox.2020.152464 PMC7270714

[35] Zoratti M; Szabo I. The mitochondrial permeability transition. Biochim. Biophys. Acta 1995, 1241, 139–176. 7640294 10.1016/0304-4157(95)00003-a

[36] Antoniel M; Giorgio V; Fogolari F; Glick GD; Bernardi P; Lippe G. The oligomycin-sensitivity conferring protein of mitochondrial ATP synthase: Emerging new roles in mitochondrial pathophysiology. Int. J. Mol. Sci 2014, 15, 7513–7536. 24786291 10.3390/ijms15057513 PMC4057687

[37] Crompton M; Ellinger H; Costi A. Inhibition by cyclosporin A of a Ca2+-dependent pore in heart mitochondria activated by inorganic phosphate and oxidative stress. Biochem. J 1988, 255, 357–360. 3196322 PMC1135230

[38] Waldmeier PC; Feldtrauer JJ; Qian T; Lemasters JJ Inhibition of the mitochondrial permeability transition by the nonimmunosuppressive cyclosporin derivative NIM811. Mol. Pharmacol 2002, 62, 22–29. 12065751 10.1124/mol.62.1.22

[39] Krauskopf A; Eriksson O; Craigen WJ; Forte MA; Bernardi P Properties of the permeability transition in VDAC1(−/−) mitochondria. Biochim. Biophys. Acta 2006, 1757, 590–595. 16626625 10.1016/j.bbabio.2006.02.007

[40] Baines CP; Kaiser RA; Sheiko T; Craigen WJ; Molkentin JD Voltage-dependent anion channels are dispensable for mitochondrial-dependent cell death. Nat. Cell Biol 2007, 9, 550–555. 17417626 10.1038/ncb1575 PMC2680246

[41] Kokoszka JE; Waymire KG; Levy SE; Sligh JE; Cai J; Jones DP; MacGregor GR; Wallace DC The ADP/ATP translocator is not essential for the mitochondrial permeability transition pore. Nature 2004, 427, 461–465. 14749836 10.1038/nature02229 PMC3049806

[42] Karch J; Bround MJ; Khalil H; Sargent MA; Latchman N; Terada N; Peixoto PM; Molkentin JD Inhibition of mitochondrial permeability transition by deletion of the ANT family and CypD. Sci. Adv 2019, 5, eaaw4597. 31489369 10.1126/sciadv.aaw4597 PMC6713508

[43] Bround MJ; Havens JR; York AJ; Sargent MA; Karch J; Molkentin JD ANT-dependent MPTP underlies necrotic myofiber death in muscular dystrophy. Sci. Adv 2023, 9, eadi2767. 37624892 10.1126/sciadv.adi2767 PMC10456852

[44] Giorgio V; von Stockum S; Antoniel M; Fabbro A; Fogolari F; Forte M; Glick GD; Petronilli V; Zoratti M; Szabo I; Dimers of mitochondrial ATP synthase form the permeability transition pore. Proc. Natl. Acad. Sci. USA 2013, 110, 5887–5892. 23530243 10.1073/pnas.1217823110 PMC3625323

[45] Carraro M; Giorgio V; Šileikytė J; Sartori G; Forte M; Lippe G; Zoratti M; Szabò I; Bernardi P Channel formation by yeast F-ATP synthase and the role of dimerization in the mitochondrial permeability transition. J. Biol. Chem 2014, 289, 15980–15985. 24790105 10.1074/jbc.C114.559633 PMC4047373

[46] Alavian KN; Beutner G; Lazrove E; Sacchetti S; Park HA; Licznerski P; Li H; Nabili P; Hockensmith K; Graham M; An uncoupling channel within the c-subunit ring of the F1FO ATP synthase is the mitochondrial permeability transition pore. Proc. Natl. Acad. Sci. USA 2014, 111, 10580–10585. 24979777 10.1073/pnas.1401591111 PMC4115574

[47] He J; Carroll J; Ding S; Fearnley IM; Walker JE Permeability transition in human mitochondria persists in the absence of peripheral stalk subunits of ATP synthase. Proc. Natl. Acad. Sci. USA 2017, 114, 9086–9091. 28784775 10.1073/pnas.1711201114 PMC5576841

[48] He J; Ford HC; Carroll J; Ding S; Fearnley IM; Walker JE Persistence of the mitochondrial permeability transition in the absence of subunit c of human ATP synthase. Proc. Natl. Acad. Sci. USA 2017, 114, 3409–3414. 28289229 10.1073/pnas.1702357114 PMC5380099

[49] Carroll J; He J; Ding S; Fearnley IM; Walker JE Persistence of the permeability transition pore in human mitochondria devoid of an assembled ATP synthase. Proc. Natl. Acad. Sci. USA 2019, 116, 12816–12821. 31213546 10.1073/pnas.1904005116 PMC6601249

[50] Pekson R; Liang FG; Axelrod JL; Lee J; Qin D; Wittig AJH; Paulino VM; Zheng M; Peixoto PM; Kitsis RN The mitochondrial ATP synthase is a negative regulator of the mitochondrial permeability transition pore. Proc. Natl. Acad. Sci. USA 2023, 120, e2303713120. 38091291 10.1073/pnas.2303713120 PMC10743364

[51] He L; Lemasters JJ Regulated and unregulated mitochondrial permeability transition pores: A new paradigm of pore structure and function? FEBS Lett. 2002, 512, 1–7. 11852041 10.1016/s0014-5793(01)03314-2

[52] He L; Lemasters JJ Heat shock suppresses the permeability transition in rat liver mitochondria. J. Biol. Chem 2003, 278, 16755–16760. 12611884 10.1074/jbc.M300153200

[53] Neginskaya MA; Solesio ME; Berezhnaya EV; Amodeo GF; Mnatsakanyan N; Jonas EA; Pavlov EV ATP Synthase C-Subunit-Deficient Mitochondria Have a Small Cyclosporine A-Sensitive Channel, but Lack the Permeability Transition Pore. Cell Rep. 2019, 26, 11–17.e12. 30605668 10.1016/j.celrep.2018.12.033 PMC6521848

[54] Neginskaya MA; Morris SE; Pavlov EV Both ANT and ATPase are essential for mitochondrial permeability transition but not depolarization. iScience 2022, 25, 105447. 36388971 10.1016/j.isci.2022.105447 PMC9647522

[55] Carrer A; Tommasin L; Šileikytė J; Ciscato F; Filadi R; Urbani A; Forte M; Rasola A; Szabò I; Carraro M; Defining the molecular mechanisms of the mitochondrial permeability transition through genetic manipulation of F-ATP synthase. Nat. Commun 2021, 12, 4835. 34376679 10.1038/s41467-021-25161-x PMC8355262

[56] Connern CP; Halestrap AP Recruitment of mitochondrial cyclophilin to the mitochondrial inner membrane under conditions of oxidative stress that enhance the opening of a calcium-sensitive non-specific channel. Biochem. J 1994, 302 Pt 2, 321–324. 7522435 10.1042/bj3020321 PMC1137230

[57] Waldmeier PC; Zimmermann K; Qian T; Tintelnot-Blomley M; Lemasters JJ Cyclophilin D as a drug target. Curr. Med. Chem 2003, 10, 1485–1506. 12871122 10.2174/0929867033457160

[58] Rehman H; Ramshesh VK; Theruvath TP; Kim I; Currin RT; Giri S; Lemasters JJ; Zhong Z NIM811 (N-methyl-4-isoleucine cyclosporine), a mitochondrial permeability transition inhibitor, attenuates cholestatic liver injury but not fibrosis in mice. J. Pharmacol. Exp. Ther 2008, 327, 699–706. 18801946 10.1124/jpet.108.143578 PMC2582973

[59] Theruvath TP; Zhong Z; Pediaditakis P; Ramshesh VK; Currin RT; Tikunov A; Holmuhamedov E; Lemasters JJ Minocycline and N-methyl-4-isoleucine cyclosporin (NIM811) mitigate storage/reperfusion injury after rat liver transplantation through suppression of the mitochondrial permeability transition. Hepatology 2008, 47, 236–246. 18023036 10.1002/hep.21912 PMC2656601

[60] Rehman H; Sun J; Shi Y; Ramshesh VK; Liu Q; Currin RT; Lemasters JJ; Zhong Z NIM811 prevents mitochondrial dysfunction, attenuates liver injury, and stimulates liver regeneration after massive hepatectomy. Transplantation 2011, 91, 406–412. 21131897 10.1097/TP.0b013e318204bdb2 PMC3399729

[61] Zhong Z; Theruvath TP; Currin RT; Waldmeier PC; Lemasters JJ NIM811, a mitochondrial permeability transition inhibitor, prevents mitochondrial depolarization in small-for-size rat liver grafts. Am. J. Transplant 2007, 7, 1103–1111. 17456198 10.1111/j.1600-6143.2007.01770.x

[62] Masubuchi Y; Suda C; Horie T Involvement of mitochondrial permeability transition in acetaminophen-induced liver injury in mice. J. Hepatol 2005, 42, 110–116. 15629515 10.1016/j.jhep.2004.09.015

[63] Reid AB; Kurten RC; McCullough SS; Brock RW; Hinson JA Mechanisms of acetaminophen-induced hepatotoxicity: Role of oxidative stress and mitochondrial permeability transition in freshly isolated mouse hepatocytes. J. Pharmacol. Exp. Ther 2005, 312, 509–516. 15466245 10.1124/jpet.104.075945

[64] Lemasters JJ Dying a Thousand Deaths: Redundant Pathways From Different Organelles to Apoptosis and Necrosis. Gastroenterology 2005, 129, 351–360. 16012960 10.1053/j.gastro.2005.06.006

[65] Kon K; Ikejima K; Okumura K; Aoyama T; Arai K; Takei Y; Lemasters JJ; Sato N Role of apoptosis in acetaminophen hepatotoxicity 19. J. Gastroenterol. Hepatol 2007, 22 (Suppl. S1), S49–S52. 17567465 10.1111/j.1440-1746.2007.04962.x

[66] Kim JS; Qian T; Lemasters JJ Mitochondrial permeability transition in the switch from necrotic to apoptotic cell death in ischemic rat hepatocytes. Gastroenterology 2003, 124, 494–503. 12557154 10.1053/gast.2003.50059

[67] Lemasters JJ; Nieminen AL; Qian T; Trost LC; Elmore SP; Nishimura Y; Crowe RA; Cascio WE; Bradham CA; Brenner DA; The mitochondrial permeability transition in cell death: A common mechanism in necrosis, apoptosis and autophagy. Biochim. Biophys. Acta 1998, 1366, 177–196. 9714796 10.1016/s0005-2728(98)00112-1

[68] Gujral JS; Knight TR; Farhood A; Bajt ML; Jaeschke H Mode of cell death after acetaminophen overdose in mice: Apoptosis or oncotic necrosis? Toxicol. Sci 2002, 67, 322–328. 12011492 10.1093/toxsci/67.2.322

[69] Jaeschke H; Williams CD; Farhood A No evidence for caspase-dependent apoptosis in acetaminophen hepatotoxicity. Hepatology 2011, 53, 718–719. 21274895 10.1002/hep.23940 PMC3058812

[70] Possamai LA; McPhail MJ; Quaglia A; Zingarelli V; Abeles RD; Tidswell R; Puthucheary Z; Rawal J; Karvellas CJ; Leslie EM; Character and temporal evolution of apoptosis in acetaminophen-induced acute liver failure*. Crit. Care Med 2013, 41, 2543–2550. 23949472 10.1097/CCM.0b013e31829791a2 PMC3939768

[71] Gunawan BK; Liu ZX; Han D; Hanawa N; Gaarde WA; Kaplowitz N c-Jun N-terminal kinase plays a major role in murine acetaminophen hepatotoxicity. Gastroenterology 2006, 131, 165–178. 16831600 10.1053/j.gastro.2006.03.045

[72] Win S; Than TA; Han D; Petrovic LM; Kaplowitz N c-Jun N-terminal kinase (JNK)-dependent acute liver injury from acetaminophen or tumor necrosis factor (TNF) requires mitochondrial Sab protein expression in mice. J. Biol. Chem 2011, 286, 35071–35078. 21844199 10.1074/jbc.M111.276089 PMC3186406

[73] Win S; Than TA; Kaplowitz N Mitochondrial P-JNK target, SAB (SH3BP5), in regulation of cell death. Front. Cell Dev. Biol 2024, 12, 1359152. 38559813 10.3389/fcell.2024.1359152 PMC10978662

[74] Win S; Than TA; Min RW; Aghajan M; Kaplowitz N c-Jun N-terminal kinase mediates mouse liver injury through a novel Sab (SH3BP5)-dependent pathway leading to inactivation of intramitochondrial Src. Hepatology 2016, 63, 1987–2003. 26845758 10.1002/hep.28486 PMC4874901

[75] Win S; Than TA; Fernandez-Checa JC; Kaplowitz N JNK interaction with Sab mediates ER stress induced inhibition of mitochondrial respiration and cell death. Cell Death Dis. 2014, 5, e989. 24407242 10.1038/cddis.2013.522 PMC4040675

[76] Win S; Than TA; Zhang J; Oo C; Min RWM; Kaplowitz N New insights into the role and mechanism of c-Jun-N-terminal kinase signaling in the pathobiology of liver diseases. Hepatology 2018, 67, 2013–2024. 29194686 10.1002/hep.29689 PMC5906137

[77] Samuvel DJ; Nguyen NT; Jaeschke H; Lemasters JJ; Wang X; Choo YM; Hamann MT; Zhong Z Platanosides, a Potential Botanical Drug Combination, Decrease Liver Injury Caused by Acetaminophen Overdose in Mice. J. Nat. Prod 2022, 85, 1779–1788. 35815804 10.1021/acs.jnatprod.2c00324 PMC9788857

[78] Jaeschke H; Bajt ML Intracellular signaling mechanisms of acetaminophen-induced liver cell death. Toxicol. Sci 2006, 89, 31–41. 16177235 10.1093/toxsci/kfi336

[79] Bajt ML; Knight TR; Lemasters JJ; Jaeschke H Acetaminophen-induced oxidant stress and cell injury in cultured mouse hepatocytes: Protection by N-acetyl cysteine. Toxicol. Sci 2004, 80, 343–349. 15115886 10.1093/toxsci/kfh151

[80] Jaeschke H. Glutathione disulfide formation and oxidant stress during acetaminophen-induced hepatotoxicity in mice in vivo: The protective effect of allopurinol. J. Pharmacol. Exp. Ther 1990, 255, 935–941. 2262912

[81] Hu J; Kholmukhamedov A; Lindsey CC; Beeson CC; Jaeschke H; Lemasters JJ Translocation of iron from lysosomes to mitochondria during acetaminophen-induced hepatocellular injury: Protection by starch-desferal and minocycline. Free Radic. Biol. Med 2016, 97, 418–426. 27345134 10.1016/j.freeradbiomed.2016.06.024 PMC4996678

[82] Nguyen NT; Du K; Akakpo JY; Umbaugh DS; Jaeschke H; Ramachandran A Mitochondrial protein adduct and superoxide generation are prerequisites for early activation of c-jun N-terminal kinase within the cytosol after an acetaminophen overdose in mice. Toxicol. Lett 2021, 338, 21–31. 33290831 10.1016/j.toxlet.2020.12.005 PMC7852579

[83] Kehrer JP; Klotz LO Free radicals and related reactive species as mediators of tissue injury and disease: Implications for Health. Crit. Rev. Toxicol 2015, 45, 765–798. 26610815 10.3109/10408444.2015.1074159

[84] Minotti G; Aust SD Redox cycling of iron and lipid peroxidation. Lipids 1992, 27, 219–226. 1326072 10.1007/BF02536182

[85] Galy B; Conrad M; Muckenthaler M Mechanisms controlling cellular and systemic iron homeostasis. Nat. Rev. Mol. Cell Biol 2024, 25, 133–155. 37783783 10.1038/s41580-023-00648-1

[86] Lane DJ; Merlot AM; Huang ML; Bae DH; Jansson PJ; Sahni S; Kalinowski DS; Richardson DR Cellular iron uptake, trafficking and metabolism: Key molecules and mechanisms and their roles in disease. Biochim. Biophys. Acta 2015, 1853, 1130–1144. 25661197 10.1016/j.bbamcr.2015.01.021

[87] McKie AT; Barrow D; Latunde-Dada GO; Rolfs A; Sager G; Mudaly E; Mudaly M; Richardson C; Barlow D; Bomford A; An iron-regulated ferric reductase associated with the absorption of dietary iron. Science 2001, 291, 1755–1759. 11230685 10.1126/science.1057206

[88] Gunshin H; Mackenzie B; Berger UV; Gunshin Y; Romero MF; Boron WF; Nussberger S; Gollan JL; Hediger MA Cloning and characterization of a mammalian proton-coupled metal-ion transporter. Nature 1997, 388, 482–488. 9242408 10.1038/41343

[89] Jenkitkasemwong S; Wang CY; Mackenzie B; Knutson MD Physiologic implications of metal-ion transport by ZIP14 and ZIP8. Biometals 2012, 25, 643–655. 22318508 10.1007/s10534-012-9526-x PMC4598647

[90] Ohgami RS; Campagna DR; Greer EL; Antiochos B; McDonald A; Chen J; Sharp JJ; Fujiwara Y; Barker JE; Fleming MD Identification of a ferrireductase required for efficient transferrin-dependent iron uptake in erythroid cells. Nat. Genet 2005, 37, 1264–1269. 16227996 10.1038/ng1658 PMC2156108

[91] Ohgami RS; Campagna DR; McDonald A; Fleming MD The Steap proteins are metalloreductases. Blood 2006, 108, 1388–1394. 16609065 10.1182/blood-2006-02-003681 PMC1785011

[92] Uchiyama A; Kim JS; Kon K; Jaeschke H; Ikejima K; Watanabe S; Lemasters JJ Translocation of iron from lysosomes into mitochondria is a key event during oxidative stress-induced hepatocellular injury. Hepatology 2008, 48, 1644–1654. 18846543 10.1002/hep.22498 PMC2579320

[93] Kurz T; Terman A; Gustafsson B; Brunk UT Lysosomes in iron metabolism, ageing and apoptosis. Histochem. Cell Biol 2008, 129, 389–406. 18259769 10.1007/s00418-008-0394-y PMC2668650

[94] Lill R. Function and biogenesis of iron-sulphur proteins. Nature 2009, 460, 831–838. 19675643 10.1038/nature08301

[95] Lill R; Hoffmann B; Molik S; Pierik AJ; Rietzschel N; Stehling O; Uzarska MA; Webert H; Wilbrecht C; Muhlenhoff U The role of mitochondria in cellular iron-sulfur protein biogenesis and iron metabolism. Biochim. Biophys. Acta 2012, 1823, 1491–1508. 22609301 10.1016/j.bbamcr.2012.05.009

[96] Braymer JJ; Freibert SA; Rakwalska-Bange M; Lill R Mechanistic concepts of iron-sulfur protein biogenesis in Biology. Biochim. Biophys. Acta Mol. Cell Res 2021, 1868, 118863. 33007329 10.1016/j.bbamcr.2020.118863

[97] Kořený L; Oborník M; Horáková E; Waller RF; Lukeš J The convoluted history of haem biosynthesis. Biol. Rev. Camb. Philos. Soc 2022, 97, 141–162. 34472688 10.1111/brv.12794

[98] Sheftel AD; Zhang AS; Brown C; Shirihai OS; Ponka P Direct interorganellar transfer of iron from endosome to mitochondrion. Blood 2007, 110, 125–132. 17376890 10.1182/blood-2007-01-068148

[99] Asano T; Komatsu M; Yamaguchi-Iwai Y; Ishikawa F; Mizushima N; Iwai K Distinct mechanisms of ferritin delivery to lysosomes in iron-depleted and iron-replete cells. Mol. Cell Biol 2011, 31, 2040–2052. 21444722 10.1128/MCB.01437-10 PMC3133360

[100] De Domenico I; Vaughn MB; Li L; Bagley D; Musci G; Ward DM; Kaplan J Ferroportin-mediated mobilization of ferritin iron precedes ferritin degradation by the proteasome. EMBO J. 2006, 25, 5396–5404. 17082767 10.1038/sj.emboj.7601409 PMC1636618

[101] De Domenico I; Ward DM; Kaplan J Specific iron chelators determine the route of ferritin degradation. Blood 2009, 114, 4546–4551. 19671920 10.1182/blood-2009-05-224188 PMC2777130

[102] Flatmark T; Romslo I Energy-dependent accumulation of iron by isolated rat liver mitochondria. Requirement of reducing equivalents and evidence for a unidirectional flux of Fe(II) across the inner membrane. J. Biol. Chem 1975, 250, 6433–6438. 808543

[103] Matlib MA; Zhou Z; Knight S; Ahmed S; Choi KM; Krause-Bauer J; Phillips R; Altschuld R; Katsube Y; Sperelakis N; Oxygen-bridged dinuclear ruthenium amine complex specifically inhibits Ca2+ uptake into mitochondria in vitro and in situ in single cardiac myocytes. J. Biol. Chem 1998, 273, 10223–10231. 9553073 10.1074/jbc.273.17.10223

[104] Zhang X; Lemasters JJ Translocation of iron from lysosomes to mitochondria during ischemia predisposes to injury after reperfusion in rat hepatocytes. Free Radic. Biol. Med 2013, 63, 243–253. 23665427 10.1016/j.freeradbiomed.2013.05.004 PMC3932485

[105] Shaw GC; Cope JJ; Li L; Corson K; Hersey C; Ackermann GE; Gwynn B; Lambert AJ; Wingert RA; Traver D; Mitoferrin is essential for erythroid iron assimilation. Nature 2006, 440, 96–100. 16511496 10.1038/nature04512

[106] Troadec MB; Warner D; Wallace J; Thomas K; Spangrude GJ; Phillips J; Khalimonchuk O; Paw BH; Ward DM; Kaplan J Targeted deletion of the mouse Mitoferrin1 gene: From anemia to protoporphyria. Blood 2011, 117, 5494–5502. 21310927 10.1182/blood-2010-11-319483 PMC3109720

[107] Nieminen AL; Schwartz J; Hung HI; Blocker ER; Gooz M; Lemasters JJ Mitoferrin-2 (Mfrn2) regulates the electrogenic mitochondrial calcium uniporter and inter-acts physically with MCU. Biophys. J 2014, 106, 581a–582a.

[108] Dietz JV; Fox JL; Khalimonchuk O Down the Iron Path: Mitochondrial Iron Homeostasis and Beyond. Cells 2021, 10, 2198. 34571846 10.3390/cells10092198 PMC8468894

[109] Aust SD; Morehouse LA; Thomas CE Role of metals in oxygen radical reactions. J. Free Radic. Biol. Med 1985, 1, 3–25. 10.1016/0748-5514(85)90025-x 3013969

[110] Dixon SJ; Lemberg KM; Lamprecht MR; Skouta R; Zaitsev EM; Gleason CE; Patel DN; Bauer AJ; Cantley AM; Yang WS; Ferroptosis: An iron-dependent form of nonapoptotic cell death. Cell 2012, 149, 1060–1072. 22632970 10.1016/j.cell.2012.03.042 PMC3367386

[111] Masaki N; Kyle ME; Farber JL tert-Butyl hydroperoxide kills cultured hepatocytes by peroxidizing membrane lipids. Arch. Biochem. Biophys 1989, 269, 390–399. 2919876 10.1016/0003-9861(89)90122-7

[112] Nieminen AL; Byrne AM; Herman B; Lemasters JJ Mitochondrial permeability transition in hepatocytes induced by t-BuOOH: NAD(P)H and reactive oxygen species. Am. J. Physiol 1997, 272, C1286–C1294. 9142854 10.1152/ajpcell.1997.272.4.C1286

[113] Adel N; Mantawy EM; El-Sherbiny DA; El-Demerdash E Iron chelation by deferasirox confers protection against concanavalin A-induced liver fibrosis: A mechanistic approach. Toxicol. Appl. Pharmacol 2019, 382, 114748. 31499193 10.1016/j.taap.2019.114748

[114] Gerson RJ; Casini A; Gilfor D; Serroni A; Farber JL Oxygen-mediated cell injury in the killing of cultured hepatocytes by acetaminophen. Biochem. Biophys. Res. Commun 1985, 126, 1129–1137. 3977907 10.1016/0006-291x(85)90303-1

[115] Byrne AM; Lemasters JJ; Nieminen AL Contribution of increased mitochondrial free Ca^2+^ to the mitochondrial permeability transition induced by tert-butylhydroperoxide in rat hepatocytes. Hepatology 1999, 29, 1523–1531. 10216138 10.1002/hep.510290521

[116] Kon K; Kim JS; Uchiyama A; Jaeschke H; Lemasters JJ Lysosomal iron mobilization and induction of the mitochondrial permeability transition in acetaminophen-induced toxicity to mouse hepatocytes. Toxicol. Sci 2010, 117, 101–108. 20584761 10.1093/toxsci/kfq175 PMC2923283

[117] Hu J; Lemasters JJ Suppression of iron mobilization from lysosomes to mitochondria attenuates liver injury after acetaminophen overdose in vivo in mice: Protection by minocycline. Toxicol. Appl. Pharmacol 2020, 392, 114930. 32109512 10.1016/j.taap.2020.114930 PMC7217634

[118] Adamson GM; Harman AW Oxidative stress in cultured hepatocytes exposed to acetaminophen. Biochem. Pharmacol 1993, 45, 2289–2294. 8517869 10.1016/0006-2952(93)90201-7

[119] Schnellmann JG; Pumford NR; Kusewitt DF; Bucci TJ; Hinson JA Deferoxamine delays the development of the hepatotoxicity of acetaminophen in mice. Toxicol. Lett 1999, 106, 79–88. 10378453 10.1016/s0378-4274(99)00021-1

[120] Kyle ME; Miccadei S; Nakae D; Farber JL Superoxide dismutase and catalase protect cultured hepatocytes from the cytotoxicity of acetaminophen. Biochem. Biophys. Res. Commun 1987, 149, 889–896. 3122747 10.1016/0006-291x(87)90491-8

[121] Kyle ME; Nakae D; Serroni A; Farber JL 1,3-(2-Chloroethyl)-1-nitrosourea potentiates the toxicity of acetaminophen both in the phenobarbital-induced rat and in hepatocytes cultured from such animals. Mol. Pharmacol 1988, 34, 584–589. 3173337

[122] Moon MS; Richie JP; Isom HC Iron potentiates acetaminophen-induced oxidative stress and mitochondrial dysfunction in cultured mouse hepatocytes. Toxicol. Sci 2010, 118, 119–127. 20667997 10.1093/toxsci/kfq230 PMC2955209

[123] Epsztejn S; Kakhlon O; Glickstein H; Breuer W; Cabantchik I Fluorescence analysis of the labile iron pool of mammalian cells. Anal. Biochem 1997, 248, 31–40. 9177722 10.1006/abio.1997.2126

[124] Kholmukhamedov A; Li L; Lindsey CC; Hu J; Nieminen AL; Takemoto K; Beeson GC; Beneker CM; McInnes C; Beeson CC; A new fluorescent sensor mitoferrofluor indicates the presence of chelatable iron in polarized and depolarized mitochondria. J. Biol. Chem 2022, 298, 102336. 35931111 10.1016/j.jbc.2022.102336 PMC9460511

[125] Chen Y; Guo X; Zeng Y; Mo X; Hong S; He H; Li J; Fatima S; Liu Q Oxidative stress induces mitochondrial iron overload and ferroptotic cell death. Sci. Rep 2023, 13, 15515. 37726294 10.1038/s41598-023-42760-4 PMC10509277

[126] Hu J; Nieminen AL; Weemhoff JL; Jaeschke H; Murphy LG; Dent JA; Lemasters JJ The mitochondrial calcium uniporter mediates mitochondrial Fe^2+^ uptake and hepatotoxicity after acetaminophen. Toxicol. Appl. Pharmacol 2023, 479, 116722. 37848124 10.1016/j.taap.2023.116722 PMC10872750

[127] Sukhbaatar N; Weichhart T Iron Regulation: Macrophages in Control. Pharmaceuticals 2018, 11, 137. 30558109 10.3390/ph11040137 PMC6316009

[128] Jaeschke H; Farhood A Neutrophil and Kupffer cell-induced oxidant stress and ischemia-reperfusion injury in rat liver. Am. J. Physiol 1991, 260, G355–G362. 2003603 10.1152/ajpgi.1991.260.3.G355

[129] Michael SL; Pumford NR; Mayeux PR; Niesman MR; Hinson JA Pretreatment of mice with macrophage inactivators decreases acetaminophen hepatotoxicity and the formation of reactive oxygen and nitrogen species. Hepatology 1999, 30, 186–195. 10385655 10.1002/hep.510300104

[130] Ju C; Reilly TP; Bourdi M; Radonovich MF; Brady JN; George JW; Pohl LR Protective role of Kupffer cells in acetaminophen-induced hepatic injury in mice. Chem. Res. Toxicol 2002, 15, 1504–1513. 12482232 10.1021/tx0255976

[131] Triantafyllou E; Pop OT; Possamai LA; Wilhelm A; Liaskou E; Singanayagam A; Bernsmeier C; Khamri W; Petts G; Dargue R; MerTK expressing hepatic macrophages promote the resolution of inflammation in acute liver failure. Gut 2018, 67, 333–347. 28450389 10.1136/gutjnl-2016-313615 PMC5868289

[132] Qiu K; Pan Y; Huang W; Li M; Yan X; Zhou Z; Qi J CXCL5 Promotes Acetaminophen-Induced Hepatotoxicity by Activating Kupffer Cells. Int. J. Mol. Sci 2023, 24, 12180. 37569554 10.3390/ijms241512180 PMC10419303

[133] Nguyen NT; Umbaugh DS; Sanchez-Guerrero G; Ramachandran A; Jaeschke H Kupffer cells regulate liver recovery through induction of chemokine receptor CXCR2 on hepatocytes after acetaminophen overdose in mice. Arch. Toxicol 2022, 96, 305–320. 34724096 10.1007/s00204-021-03183-0 PMC8762790

[134] Starke PE; Farber JL Ferric iron and superoxide ions are required for the killing of cultured hepatocytes by hydrogen peroxide. Evidence for the participation of hydroxyl radicals formed by an iron-catalyzed Haber-Weiss reaction. J. Biol. Chem 1985, 260, 10099–10104. 2991275

[135] Gores GJ; Flarsheim CE; Dawson TL; Nieminen AL; Herman B; Lemasters JJ Swelling, reductive stress, and cell death during chemical hypoxia in hepatocytes. Am. J. Physiol 1989, 257, C347–C354. 2764095 10.1152/ajpcell.1989.257.2.C347

[136] Farber JL; Leonard TB; Kyle ME; Nakae D; Serroni A; Rogers SA Peroxidation-dependent and peroxidation-independent mechanisms by which acetaminophen kills cultured rat hepatocytes. Arch. Biochem. Biophys 1988, 267, 640–650. 3214174 10.1016/0003-9861(88)90072-0

[137] Yamada N; Karasawa T; Kimura H; Watanabe S; Komada T; Kamata R; Sampilvanjil A; Ito J; Nakagawa K; Kuwata H; Ferroptosis driven by radical oxidation of n-6 polyunsaturated fatty acids mediates acetaminophen-induced acute liver failure. Cell Death Dis. 2020, 11, 144. 32094346 10.1038/s41419-020-2334-2 PMC7039960

[138] Stockwell BR; Friedmann Angeli JP; Bayir H; Bush AI; Conrad M; Dixon SJ; Fulda S; Gascon S; Hatzios SK; Kagan VE; Ferroptosis: A regulated cell death nexus linking metabolism, redox biology, and disease. Cell 2017, 171, 273–285. 28985560 10.1016/j.cell.2017.09.021 PMC5685180

[139] Shi Y; Xu N; Liu B; Ma Y; Fu X; Shang Y; Huang Q; Yao Q; Chen J; Li H Mifepristone protects acetaminophen induced liver injury through NRF2/GSH/GST mediated ferroptosis suppression. Free Radic. Biol. Med 2024, 222, 229–243. 38906233 10.1016/j.freeradbiomed.2024.06.014

[140] Tao J; Xue C; Wang X; Chen H; Liu Q; Jiang C; Zhang W GAS1 Promotes Ferroptosis of Liver Cells in Acetaminophen-Induced Acute Liver Failure. Int. J. Med. Sci 2023, 20, 1616–1630. 37859699 10.7150/ijms.85114 PMC10583184

[141] Hinson JA; Pike SL; Pumford NR; Mayeux PR Nitrotyrosine-protein adducts in hepatic centrilobular areas following toxic doses of acetaminophen in mice. Chem. Res. Toxicol 1998, 11, 604–607. 9625727 10.1021/tx9800349

[142] Knight TR; Kurtz A; Bajt ML; Hinson JA; Jaeschke H Vascular and hepatocellular peroxynitrite formation during acetaminophen toxicity: Role of mitochondrial oxidant stress. Toxicol. Sci 2001, 62, 212–220. 11452133 10.1093/toxsci/62.2.212

[143] Cover C; Mansouri A; Knight TR; Bajt ML; Lemasters JJ; Pessayre D; Jaeschke H Peroxynitrite-induced mitochondrial and endonuclease-mediated nuclear DNA damage in acetaminophen hepatotoxicity. J. Pharmacol. Exp. Ther 2005, 315, 879–887. 16081675 10.1124/jpet.105.088898

[144] Campolo N; Bartesaghi S; Radi R Metal-catalyzed protein tyrosine nitration in biological systems. Redox Rep. 2014, 19, 221–231. 24977336 10.1179/1351000214Y.0000000099 PMC6837402

[145] Du K; Farhood A; Jaeschke H Mitochondria-targeted antioxidant Mito-Tempo protects against acetaminophen hepatotoxicity. Arch. Toxicol 2017, 91, 761–773. 27002509 10.1007/s00204-016-1692-0 PMC5033665

[146] Esterbauer H; Eckl P; Ortner A Possible mutagens derived from lipids and lipid precursors. Mutat. Res 1990, 238, 223–233. 2342513 10.1016/0165-1110(90)90014-3

[147] Esterbauer H; Cheeseman KH Determination of aldehydic lipid peroxidation products: Malonaldehyde and 4-hydroxynonenal. Methods Enzymol. 1990, 186, 407–421. 2233308 10.1016/0076-6879(90)86134-h

[148] Esterbauer H; Schaur RJ; Zollner H Chemistry and biochemistry of 4-hydroxynonenal, malonaldehyde and related aldehydes. FreeRadic. Biol. Med 1991, 11, 81–128. 10.1016/0891-5849(91)90192-6 1937131

[149] Wendel A; Feuerstein S; Konz KH Acute paracetamol intoxication of starved mice leads to lipid peroxidation in vivo. Biochem. Pharmacol 1979, 28, 2051–2055. 475847 10.1016/0006-2952(79)90223-5

[150] Wendel A; Feuerstein S Drug-induced lipid peroxidation in mice--I. Modulation by monooxygenase activity, glutathione and selenium status. Biochem. Pharmacol 1981, 30, 2513–2520. 7306203 10.1016/0006-2952(81)90576-1

[151] Wendel A; Jaeschke H; Gloger M Drug-induced lipid peroxidation in mice--II. Protection against paracetamol-induced liver necrosis by intravenous liposomally entrapped glutathione. Biochem. Pharmacol 1982, 31, 3601–3605. 6295406 10.1016/0006-2952(82)90582-2

[152] Knight TR; Fariss MW; Farhood A; Jaeschke H Role of lipid peroxidation as a mechanism of liver injury after acetaminophen overdose in mice. Toxicol. Sci 2003, 76, 229–236. 12944590 10.1093/toxsci/kfg220

[153] Younes M; Cornelius S; Siegers CP Ferrous ion supported in vivo lipid peroxidation induced by paracetamol--its relation to hepatotoxicity. Res. Commun. Chem. Pathol. Pharmacol 1986, 51, 89–99. 3952373

[154] Albano E; Poli G; Chiarpotto E; Biasi F; Dianzani MU Paracetamol-stimulated lipid peroxidation in isolated rat and mouse hepatocytes. Chem. Biol. Interact 1983, 47, 249–263. 6652811 10.1016/0009-2797(83)90161-8

[155] Minamide Y; Horie T; Tomaru A; Awazu S Spontaneous chemiluminescence production, lipid peroxidation, and covalent binding in rat hepatocytes exposed to acetaminophen. J. Pharm. Sci 1998, 87, 640–646. 9572917 10.1021/js9701014

[156] Wimborne HJ; Hu J; Takemoto K; Nguyen NT; Jaeschke H; Lemasters JJ; Zhong Z Aldehyde dehydrogenase-2 activation decreases acetaminophen hepatotoxicity by prevention of mitochondrial depolarization. Toxicol. Appl. Pharmacol 2020, 396, 114982. 32240663 10.1016/j.taap.2020.114982 PMC7716617

[157] Chen CH; Budas GR; Churchill EN; Disatnik MH; Hurley TD; Mochly-Rosen D Activation of aldehyde dehydrogenase-2 reduces ischemic damage to the heart. Science 2008, 321, 1493–1495. 18787169 10.1126/science.1158554 PMC2741612

